# Ontario Veterinary College Medical Society

**Published:** 1892-03

**Authors:** J. S. Grove

**Affiliations:** Secretary


*Ontario Veterinary College Medical Society.*—The students of the Ontario Veterinary College are taking great interest in the meetings which they hold on Tuesday and Friday evenings of each week. These gatherings prove of infinite benefit to the students, indeed, the Society is one of the strong points in the college course, for where so many students are met together from all parts of the continent, their combined experience is of great benefit to the members, and we are pleased to note that the students all show that true professional spirit which is expressed in the words, "There are secrets among us."

The essays which have been read thus far in the session have been unusually well prepared, and what is specially encouraging, is the fact that the essayists show an unusual inclination to investigate their subjects for themselves, and not rely wholly on what is laid down in the text books. Among the papers recently read are the following : E. G. Kriebel, Herford, Pa., "Cerebro Spinal Meningitis." Mr. Kriebel gave a very nice account of what he had observed in regard to the causes and symptoms of this malady. J. S. Elliott, Gasport, N. Y., "Abortion." Mr. Elliott related some very interesting experiments, and showed that this grave disorder could be overcome by the proper and persistent use of antiseptics. R. H Fortune, Chesley, Ont., "Breeding." This paper provoked a very prolonged discussion, during which many interesting and amusing anecdotes were related. Among the interesting cases which have been reported are the following : W. E. Stocking, Eagle Harbor, N. Y., reports the case of a badly glandered mare which suckled her colt for several months, and the colt remained free from the disease. P. Gaunt, Detroit, Mich., reports a case of tetanus resulting from a wound in the foot. Neurotomy was resorted to on the affected limb, and with the result that the spasms almost immediately subsided, and the wound healed nicely in due time.

J. S. GROVE, *Secretary.*

